# Atoning Past Indulgences: Oral Consumption and Moral Compensation

**DOI:** 10.3389/fpsyg.2019.02103

**Published:** 2019-09-13

**Authors:** Thea S. Schei, Sana Sheikh, Simone Schnall

**Affiliations:** ^1^Department of Psychology, University of Cambridge, Cambridge, United Kingdom; ^2^Department of Psychiatry, Cambridge Health Alliance, Cambridge, MA, United States; ^3^Harvard Medical School, Boston, MA, United States

**Keywords:** morality, eating, moral compensation, guilt, self-silencing, prosocial behavior, cold pressor, punishment

## Abstract

Previous research has shown that moral failures increase compensatory behaviors, such as prosociality and even self-punishment, because they are strategies to re-establish one’s positive moral self-image. Do similar compensatory behaviors result from violations in normative eating practices? Three experiments explored the moral consequences of recalling instances of perceived excessive food consumption. In Experiment 1 we showed that women recalling an overeating (vs. neutral) experience reported more guilt and a desire to engage in prosocial behavior in the form of so-called self-sacrificing. In Experimental 2 this logic was applied to actual spontaneous helping behaviors toward an experimenter, with participants who recalled an overeating (vs. neutral) experience exhibiting more such helping in the laboratory. Experimental 3 expanded the investigation to self-inflicted pain: overeating (vs. neutral) recall led to higher levels of self-punishment as indicted by longer time periods spent engaging in the cold pressor task. In sum, failures in normative food consumption can be viewed as moral transgressions that elicits both interpersonal and intrapersonal compensatory behaviors aimed at restoring a positive moral self-image.

## Introduction

Food consumption is not merely a biological necessity but carries social and moral significance (Stein and Nemeroff, 1995; Rozin, 1996; Sheikh et al., 2013). Indeed, the moral connotation of eating is reflected in everyday food language, for example, when describing a chocolate cake as “sinful” and vegetable juice as “purifying.” Do such linguistic expressions reflect a link between food consumption and morality? And, if so, what are the behavioral consequences of viewing food consumption as a moral or immoral behavior? The present studies explored the moral nature of eating by investigating its influence on moral emotional and behavioral responses. In particular, we tested whether remembering excessive food consumption as a moral violation invokes compensatory behaviors in the moral realm as a way to “redeem” oneself.

Morality involves value judgments of right and wrong, good and bad, normative and non-normative, regarding one’s actions and the actions of others. Although food consumption at the most basic, biological level functions to nourish the body, historically it has been imbued with social and cultural practices. In Ancient Greece, Plato advocated moderate consumption because he reasoned that the body constrains the soul and thus prevents access to “universal truths” [Plato’s Phaedo in Gallop (1993)]. In the Middle Ages women who are now known as “holy anorexics” claimed to abstain from food, sometimes for decades, to come closer to God (Reda and Sacco, 2001). At a lesser extreme, many religions to this day prescribe followers to adhere to certain consumption rules. For example, eating meat is forbidden in certain Buddhist and Hindu groups, and pork in particular in Judaism and Islam. Furthermore, across religions fasting has been used to reach enlightenment, and deliverance from sin. Indeed, a long history of attaching moral value to food consumption exists among different religious groups across historical and cultural contexts (Coveney, 2006).

The moralization of eating is not unique to religious groups, however. Secular perspectives on eating practices are becoming increasingly prevalent, particularly ones that value restraining the type and amount of food one consumes (Herman and Polivy, 1980). Reflected in the popularity of fad dieting, “detoxes,” and the prevalence of eating disorders (Bundros et al., 2016; Dunn and Bratman, 2016), one’s sense of being “good” or “bad” is intimately tied to restrained eating practices, both in Western societies and increasingly around the world. Thus, eating practices as strategies to connect with the divine are no longer as ubiquitous, but eating is nevertheless often pursued as a way to achieve moral purity by keeping the body clean and treating it “as a temple” (Saguy and Gruys, 2010).

The experiences of food consumption converge on the commonly expressed belief that “you are what you eat” (Nemeroff and Rozin, 1989; Vartanian et al., 2007). People eating a healthy meal are seen as more kind-hearted and virtuous (Stein and Nemeroff, 1995) and more moral (Oakes and Slotterback, 2004–2005). Those with a slimmer figure are judged as “good” and “virtuous” (Bordo, 1993), whereas having a larger body size is “bad” and “gluttonous” (Crandall and Eshleman, 2003; Latner and Stunkard, 2003). Individuals with eating disorders tend to categorize food as morally “good” or “bad” depending on its perceived healthiness (Bauer et al., 1986; Johnson et al., 1987) and they associate restricted eating with physical purity (Skårderud, 2007). Thus, across time and geographical locations, in religious as well as in secular societies, food-related practices take on moral properties to be praised or condemned.

Regarding affective experience, shame and guilt are the quintessential emotions related to one’s sense of morality (Baumeister et al., 1994). Although both shame and guilt involve self-blame for a transgression, the two are largely differentiated by their attributional styles: Guilt involves a negative evaluation of a specific action (e.g., “I did something bad”) whereas shame involves a globally negative evaluation of one’s entire self (e.g., “I am a bad person”) (Tangney et al., 2007). Whereas guilt reflects a temporarily compromised moral self, for shame the defects in the self is felt to be stable and permanent (Tracy and Robins, 2006).

Shame and guilt are also relevant in perceived failures of eating. Women with eating disorders display higher levels of guilt and shame related to food consumption than women without such disorders (Sanftner et al., 1995; Burney and Irwin, 2000). Moreover, a large proportion of the Western population in some way restrains food intake, for example, by being on a “diet” (Hill, 2002; Fayet et al., 2012). Indeed, dieters associate high-calorie foods with guilt (Fletcher et al., 2007; Kuijer and Boyce, 2014) and report feelings of guilt after eating chocolate (Macht and Dettmer, 2006). College women also report feelings of guilt after eating snacks such as candy and ice cream (Steenhuis, 2009). Even the mere recall of an overeating event (Sheikh et al., 2013; Piqueras-Fiszman and Jaeger, 2016) can elicit guilt, shame, anger and disgust. This indicates that non-normative eating practices, and particularly failures in restrained eating, evoke emotional reactions typical of moral transgressions.

If non-normative eating practices induce emotions associated with moral transgressions, then we would expect failures in normative eating to elicit the same behavioral tendencies as typical moral transgressions. Indeed, people have a fundamental need to view themselves as moral beings (Steele, 1988; Sherman and Cohen, 2006) and moral character is a key concern when forming impressions about others (Willis and Todorov, 2006; Brambilla et al., 2011). On an implicit level people appear to monitor their moral value, as if accumulating “moral credits” when doing something good and later “cashing in” these credits when doing something bad (Merritt et al., 2010; Mullen and Monin, 2016; for a meta-analysis, see Blanken et al., 2015).

In contrast, moral failures deplete “moral credits” and elicit guilt and shame, which threatens one’s favorable self-image and motivating attempts to “make up” for one’s wrongdoings by engaging in praiseworthy behavior (e.g., Roseman et al., 1994; Tangney et al., 2007). In their seminal paper, Carlsmith and Gross (1969) showed that after delivering painful shocks to a confederate, participants were more likely to assist with a subsequent request. Likewise, after hurting another person, people engaged in confessing, apologizing, and making concessions to restore jeopardized relationships as well as their moral self-image (Schlenker and Darby, 1981; Ohbuchi et al., 1989; Gonzales et al., 1992). Moral transgressions also activate reparative behaviors toward others besides the specific victim of the transgression. For example, non-cooperation in one round of a social bargaining game increased cooperation in an unrelated round (Ketelaar and Au, 2003). Similarly, recalling a past immoral action increased likelihood of helping the experimenter (Ding et al., 2016), cheating less on a future test (Jordan et al., 2011), and donating more time (Stone et al., 1997) or money to charity (Jordan et al., 2011).

Moral failures not only increase compensatory behaviors such as prosocial actions, but also efforts to alleviate one’s guilt through self-punishment (Nelissen and Zeelenberg, 2009), including agreeing to receive physical pain (Nelissen, 2011) and inflicting pain oneself. Bastian et al. (2011) found that after recalling a time they socially excluded another person, participants spent more time immersing their arms in painfully cold water compared to those who recalled a neutral memory. Moreover, this self-punishment reduced guilt. Participants also administered stronger electric shocks to themselves after writing about a guilt-inducing event than after a neutral event (Inbar et al., 2012), which further suggests that painful self-punishment functions to re-establish one’s own moral self-image and gather “moral credits.”

Finally, several studies also found that being confronted with one’s moral transgressions increases the need to engage in physical cleansing, as if to symbolically “wash off the sin” (for reviews, see West and Zhong, 2015; Lee and Schwarz, 2016). Zhong and Liljenquist (2006) found that after recalling an unethical (vs. ethical) deed, participants completed word fragments with more cleansing related words and chose antiseptic wipes—a cleansing product—over a pen. Moreover, participants who washed their hands after recalling an unethical deed were less likely to indicate an intention to volunteer than those who did not wash their hands, suggesting that washing restored their sense of morality.^1^ The findings converge on a general “clean slate” effect, whereby cleaning behaviors reduces threats to the self by creating psychological separation between the person and the threat (Lee and Schwarz, 2016). All in all, the research indicates that both acting immorally and recalling immoral behavior increases direct as well as indirect restitution as part of a general “moral credits” phenomenon.

Given the moralization of eating practices and its relationship to guilt, failures in healthy and restrained eating may also increase efforts in moral compensation. Indeed, Sheikh et al. (2013) found that women who recalled a time they overate were more likely to complete ambiguous word-fragments with cleanliness-related words, but no such effect occurred for men (Study 1). After such recall women also expressed a preference for a cleansing wipe or hand-gel over a pen (Study 2). The effect of gender is likely due to the fact that the thin ideal and eating healthy is a more important part of having a high social appeal for women than for men (Vartanian et al., 2007). Mirroring the findings of moral cleansing to “wash away one’s sins” (Zhong and Liljenquist, 2006), these findings suggest that failures in restrained eating also increase motivation to reestablish one’s moral self-image. In this paper, we tested whether recalling act of excessive food consumption also leads to compensatory behavior, namely with respect to first, prosocial behavior, and second, self-punishment.

Although morality in the context of eating healthily is pervasive, very little is known about the consequences of this link for the individual. Across three experiments we investigated the consequences of failures in normative eating practices behaviors on feelings of guilt and efforts in moral compensation. Experiment 1 investigated whether remembering an overeating (vs. neutral) event leads to increased guilt and therefore, more reported prosocial behavior in the form of sacrificing one’s own needs for the benefit of others, what is known as *self-silencing*. Experiment 2 tested whether this effect increases actual prosocial behaviors. Finally, Experiment 3 examined whether remembering an overeating (vs. neutral) event increases moral compensation in the form of self-punishment. Previous research (Sheikh et al., 2013) found women, but not men, to engage in moral cleansing behaviors after recalling an overeating memory—presumably because moral norms encouraging food restriction are more pronounced for them (Vartanian et al., 2007). Furthermore, because the experimenter was female it is possible that male participants may exhibit greater helping behavior (Eagly and Crowley, 1986) or pain-endurance (Fowler et al., 2011) to demonstrate their masculinity. Therefore, the experiments included only women. Taken together, the research explored the emotional and behavioral consequences of the moralization of eating in the population for which it likely matters most, namely women. We report all manipulations and measures, how we arrived at sample sizes, and whether any participants were excluded from analysis.

## Experiment 1

We first explored the link between normative food consumption and prosociality by testing whether reminders of excessive eating in the past would lead to a greater propensity to report moral behavior in the form of so-called *self-silencing*, which refers to the tendency of putting the needs of others before one’s own, often as a strategy to build and maintain interpersonal relationships (Jack, 1991). Self-silencing here is considered a type of prosociality because it is a tendency that is costly to the self and benefits someone else (Eisenberg, 1982). Furthermore, self-silencing has been considered a negative form of prosociality often carried out by women (Harway and Nutt, 2006) because although it is intended to benefit others, it nevertheless has negative consequences for the wellbeing of the self-silencer due to the withdrawal of personal needs. It is also especially prevalent in women with disordered eating (e.g., Buchholz et al., 2007; Hambrook et al., 2010; Norwood et al., 2011; Shouse and Nilsson, 2011), which is thought to occur because both involve the denial of one’s own personal desires. Furthermore, placing importance on both self-silencing as well as restrained eating falls under the rubric of striving to become a traditionally “good woman.” Because self-silencing entails the giving up of one’s own needs and desires for the benefit of others, we hypothesized that after having thought of a past instance when they engaged in excessive eating, women should indicate higher levels of self-silencing. This method was chosen because recalling a past event as a tool to activate moral psychological processes has been used extensively in moral psychological research (e.g., Stone et al., 1997; Zhong and Liljenquist, 2006; Jordan et al., 2011; Sheikh et al., 2013; Ding et al., 2016). Following Sheikh et al. (2013) manipulation we asked participants to recall and describe in detail a time they “ate too much” or in the control condition, “your typical journey to work/place of study.” Because guilt and shame are especially relevant given their status as emotions associated with prosociality and compensation, and withdrawal and self-attack respectively (Tangney et al., 2007), we predicted that relative to participants recalling a neutral event those recalling an overeating event would report an increased tendency to self-silence, with a mediating role of guilt and shame.

### Methods

#### Participants

One hundred and sixty-three female students from the University of St Andrews and the University of Cambridge were recruited through online advertisements and the university’s research recruitment service for an online study with the chance of winning a £20 Amazon voucher. They had a mean age of 22.63 years (*SD* = 5.66) and reported their ethnicities to be White (69.2%), Asian (20.7%), Mixed (3.0%), and Other (1.2%). Ten participants did not report their ethnicity. A preliminary correlational study (*n* = 63) investigating the association between restrained eating and self-silencing indicated a medium effect size *r* = 0.35. Using G^∗^Power we calculated the sample size for a one-tailed *t*-test using a medium effect size of Cohen’s *d* = 0.50, a *p*-value of 0.05 and statistical power of 0.90. This returned a sample size of 140. We intended to stop at this number but due to practicalities with the data collection we ended up with a somewhat larger and more highly powered sample, namely 160 participants.

#### Procedure

Participants were recruited for an online study on eating behaviors and emotions, using the testing platform Qualtrics. The random assignment function on Qualtrics was used to assign participants to one of the two conditions. After giving electronic informed consent participants completed a recall task and then the State Shame and Guilt Scale, the Silencing-the-Self-Scale and the Dutch Eating Behavior Questionnaire. Subsequently participants were debriefed, thanked and entered into the prize draw.

#### Manipulation

Participants were randomly assigned to recall a specific time they overate or a neutral memory, with instructions taken from Sheikh et al. (2013) and Schnall et al. (2010), respectively. The overeating recall instructed participants to “please think back to a time you *ate too* much and describe this experience in as much detail as possible,” while the neutral recall read “please think of your typical journey to *work/place of study* and describe this experience in as much detail as possible.” We chose these instructions due to the variation in what people might consider overeating. It was important that participants felt the memory they recalled contained an episode they construed as overeating. For example, giving a direct instruction such as “eating a piece of chocolate cake” could be interpreted differently from participant to participant, while leaving it up to participants which example to consider was more effective and also ecologically valid. Participants were asked to be as detailed as possible, and although as in earlier research (Zhong and Liljenquist, 2006; Schnall et al., 2010; Sheikh et al., 2013; Gilchrist and Schnall, 2018) no minimum word requirement was specified, all content was checked to ascertain that the recall produced by participants conformed to the instructions. Indeed, all participants provided relevant narratives, describing either a food- or eating-related memory for the overeating recall instructions, and a typical journey for the neutral recall instructions.

#### Measures

##### Silencing the self scale (Jack and Dill, 1992)

With 31-items the scale assesses participants’ tendency to put the need of others before their own. It has four subscales: tendency to focus on others’ perceptions of oneself (Externalized Self-Perception), tendency to view others’ needs as more important than one’s own (Care as Self-Sacrifice), tendency to suppress self-expression (Silencing the Self), and exhibiting discrepancy between one’s true self and public self-image (Divided Self). Items include, “I tend to judge myself by how I think other people see me” (Externalized Self-Perception), “Considering my needs to be as important as those of the people I love is selfish” (Care-as-Self-Sacrifice), “I rarely express my anger at those close to me” (Silencing the Self), and “When I am in a close friendship I lose my sense of who I am” (Divided Self), rated from 1 (“strongly disagree”) to 5 (“strongly agree”). Because we used a student sample the original self-silencing scale was amended by replacing mentions of romantic relationships with close friendships, as suggested by Sippola and Bukowski (1996). Mean scores are used with higher scores indicating larger extent of self-silencing.

##### State shame and guilt scale (Marschall et al., 1994)

The scale measures current feelings of shame, guilt, and pride, with items such as “I want to sink into the floor and disappear” for shame (α = 0.87), or “I feel like apologizing, confessing” for guilt (α = 0.89), rated from 1 (“not feeling this way at all”) to 5 (“feeling this way very strongly”). Mean scores are used with higher scores representing stronger feelings of shame and guilt. Pride was not of interest and responses were only included as fillers.

##### Dutch eating behavior questionnaire (van Strien et al., 1986)

As a potential moderator the restrained eating subscale of the Dutch Eating Behavior Questionnaire was used to measure restrained eating tendency. This measure was chosen over other common measures of dietary restraint such as the Revised Restraint Scale (Herman and Polivy, 1975) because it does not contain references to emotions associated with overeating, and does not require the participants to specify weight loss in numbers, which has been found to decrease completion rates (Wardle, 1985). The scale consists of 11 items (α = 0.93), such as “Do you take into account your weight with what you eat?” and “Do you eat less at meal times than you would like to eat?” Participants are asked to answer each question on a scale from 1 (“Never”) to 5 (“Very Often”). Mean scores are used with higher scores indicating a larger degree of eating restraint. The measure also includes a question asking whether the participant has had, or currently has, an eating disorder (Anorexia Nervosa, Bulimia Nervosa and Binge-eating Disorder). Controlling for eating disorder status did not change the results, so to maximize statistical power all participants were retained.

##### Bem sex role inventory (Bem, 1974)

To measure identification with traditionally feminine or masculine gender roles, participants rated themselves on 60 characteristics such as “compassionate,” “gentle” (α = 0.80), or “self-sufficient” and “competitive” (α = 0.85) from 1 (“Never or almost never true”) to 7 (“Almost always true”). The Bem Sex Role Inventory was included as an exploratory measure only. No reported analyses include the measure.

### Results

**FIGURE 1:**
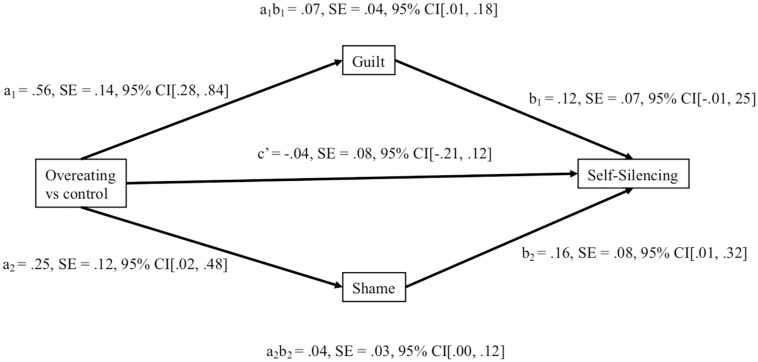
Parallel multiple mediation model showing the indirect effect of recalled overeating memory on self-silencing through guilt and shame in Experiment 1.

An independent *t*-test examined whether recalling an overeating instance made participants more likely to report self-silencing behavior. Contrary to expectations, there was no difference between the overeating (*M* = 2.70, *SD* = 0.56) and control groups (*M* = 2.76, *SD* = 0.54), *t* (161) = 0.76, *p* = 0.45, $\eta_{\text{p}}^{2}$ = 0.004, 95% CI [−0.24, 0.11]. However, given that mediation models can show indirect effects despite the absence of a direct effect (Zhao et al., 2010; Hayes, 2013), it is possible that only participants experiencing guilt or shame as a result of the overeating memory would report higher self-silencing. Indeed, an independent *t*-test showed a difference between the two groups for guilt (overeating: *M* = 2.33, *SD* = 0.93; control: *M* = 1.77, *SD* = 0.88), *t* (165) = 4.02, *p* < 0.001, $\eta_{\text{p}}^{2}$ = 0.09, 95% CI [0.29, 0.84], with a regression showing that guilt in turn predicted self-silencing, *F* (1,161) = 0.24.71, *p* < 0.001, $\eta_{\text{p}}^{2}$ = 0.133, 95% CI [2.65, 2.81]. The same pattern occurred for shame, with the overeating group reporting more shame (*M* = 1.96, *SD* = 0.85) than the control group (*M* = 1.70, *SD* = 0.66), *t* (157.63) = 2.24, *p* = 0.03, $\eta_{\text{p}}^{2}$ = 0.03, 95% CI [0.03, 0.50], with a regression showing that shame predicted self-silencing, *F* (1, 161) = 25.98, *p* < 0.001, $\eta_{\text{p}}^{2}$ = 0.13, 95% CI [2.65, 2.81]. Adjusted degrees of freedom are reported because unequal variances were found, *F* = 4.93, *p* = 0.03. Therefore, we conducted parallel multiple mediational analyses [Model 4 in Hayes (2013); see Figure 1]. The model showed a total indirect effect via state guilt and shame, β = 0.11, *SE* = 0.04, 95% CI [0.03, 21], with both state guilt and state shame displaying separate indirect effects.

To test whether restrained eating tendency affected the influence of the overeating recall on reported self-silencing, restrained eating was entered as a moderator of the direct effect between the overeating manipulation and the self-silencing report. Because guilt and shame mediated this effect, restrained eating was also entered as a moderator of the mediation effect, by use of Model 8 (Hayes, 2013). The model showed that restrained eating moderated the direct effect of overeating recall on self-silencing, β = 0.17, SE = 0.08, *t* = 2.04, *p* = 0.04, 95% CI [0.01, 33]. However, there was no moderation of the indirect effect through guilt by restrained eating, index = −0.02, *SE* = 0.03, 95% CI [−0.09, 0.02], or for guilt, index = 0.00, *SE* = 0.02, 95% CI [−0.03, 0.04].

### Discussion

Although Experiment 1 did not find a direct effect of remembering excessive consumption on self-silencing, it demonstrated that the effect was mediated by feelings of guilt and shame. Recalling an episode of having overeaten resulted in increased guilt and shame, which in turn mediated the relationship with self-silencing, such that participants experiencing guilt were more likely to report self-silencing. This provided further support for the established link between restrained eating and self-silencing (e.g., Hambrook et al., 2010; Norwood et al., 2011; Shouse and Nilsson, 2011). Contrary to our hypothesis, we did not find that recalling an overeating episode directly led to higher self-silencing. Thus, it may not be sufficient to merely reflect on previous overindulgences, but additionally it is necessary to experience guilt as a consequence.

Overall, Experiment 1 showed the self-silencing consequences of the moralization of restrained eating in women, thereby highlighting the potential real-life consequences of the moral nature of eating. Our findings suggest that self-silencing is a form of moral compensation that can be elicited by even the mere reminder of a restrained eating failure. One limitation of this experiment is the reliance on self-report instead of actual prosocial behavior, and thus, Experiment 2 employed a measure of actual voluntary helping.

## Experiment 2

As discussed above, moral transgressions often lead to greater efforts in moral compensation, often via prosocial behavior such as helping (Carlsmith and Gross, 1969; Stone et al., 1997; Ding et al., 2016). In Experiment 2 we therefore, assessed voluntary helping using a paradigm developed by Bartlett and DeSteno (2006) and subsequently used by Schnall et al. (2010), which involved helping the experimenter with a tedious task. Participants were given the chance to help after completing the same memory recall as used in Experiment 1. We predicted that recounting an overeating (vs. neutral) memory would increase the time spent helping the experimenter.

### Methods

#### Participants

A total of 63 female students from the University of Cambridge between the ages of 18 and 35 (*M* = 21.7, *SD* = 3.16) participated in exchange for monetary compensation. 71% identified as White, 15% as East-Asian, 4% as Asian-Indian, 6% as Mixed; 4% did not disclose their ethnicity. One participant withdrew from the study before completion, and two participants were removed due to guessing the purpose of the study, leaving a sample of 60. An *a priori* sample size calculation was carried out with the “pwr” package in R. The calculation was based on Study 2 from Schnall et al. (2010), which had an effect size of $\eta_{\text{p}}^{2}$ = 0.32. Due to the small sample size of this study we increased the power and significance level to arrive at a more precise effect size estimate. An $\eta_{\text{p}}^{2}$ = 0.32, with, 0.99 power at a 0.01 level of significance returned a sample of 60.

#### Procedure

A female experimenter tested participants individually in a laboratory. Participants’ assignment to conditions was alternated throughout the study period. They were told that the study concerned episodic memory. Importantly, it was specified at recruitment that the study would last 1 h and that payment was commensurate with this duration. After the recall task participants went on to complete the ostensible episodic memory task on the computer. When the task did not launch due to a technical error, participants were paid and told they were free to leave. Upon gathering their things, they were offered the chance to help the experimenter with a separate task consisting of completing tedious math questions. It was emphasized to participants that there was no obligation to help (see Schnall et al., 2010, for full procedure). If they agreed they received the rather large pack with the math problems. At the end participants were probed for suspicion by being asked what the thought was the purpose of the study. Any mention of the relationship between morality and eating, emotional consequences of eating or compensation after eating was used to identify awareness of the study aims. None reported any insights. Then they were debriefed, compensated and thanked.

#### Manipulation

Participants were assigned to one of the groups used in Experiment 2. They had 10 min to write down a memory while the experimenter waited outside the room. The experimenter returned to the room once the allotted time was up.

#### Measures

##### Helping behavior

As in Bartlett and DeSteno (2006) and Schnall et al. (2010) we recorded the seconds that participants spent on a booklet consisting of 65 mathematical questions, which was estimated to take no longer than the study session (40 min).

##### Liking of mathematics

To rule out a potential confound we also assessed enjoyment of mathematics on a scale from 1(“Not at all”) to 7 (“Very much so”).

### Results

#### Liking of Mathematics

An independent *t*-test showed no difference between the two groups (overeating *M* = 4.05, *SD* = 1.59, control *M* = 4.26, *SD* = 4.26) in terms of the liking of mathematics, *t* (55) = 0.53, *p* = 0.59, *d* = 0.20, 95% CI [−0.58, 0.99].

#### Questionnaire Completion Time

No outliers in the distribution of response times were found. An independent samples *t*-test tested whether participants who recalled an overeating memory would help for longer than those who recalled a neutral event. As predicted, the overeating group (*M* = 38.03, *SD* = 12.13) spent significantly more time on the mathematics questionnaire than the neutral group (*M* = 25.76, *SD* = 14.20), t (58) = 3.60, *p* < 0.001, *d* = 0.94, 95% CI [5.42, 19.13].^2^

#### Number of Completed Items

It could be that increased completion time in the overeating group was not a reflection of helping, but due to exhaustion after recalling an emotional memory. Indeed, recalling an overeating event has previously been shown to elicit negative feelings such as guilt, shame, disgust, and anger at the self (see Sheikh et al., 2013), and negative emotions can impair participants’ working memory and problem solving capacity (Cavalera and Pepe, 2014). However, the overeating group (items attempted *M* = 52.80, *SD* = 11.34, correct items *M* = 40.30, *SD* = 10.65) both tackled more items, *t* (58) = 3.05, *p* = 0.004, *d* = 0.81, 95% CI [3.54, 17.08] and answered more items correctly, *t* (58) = 2.62, *p* = 0.01, *d* = 0.70, 95% CI [1.86, 14.06] than the control group (items attempted *M* = 41.48, *SD* = 14.69, correct items *M* = 31.33, *SD* = 12.29).

### Discussion

Experiment 2 demonstrated that participants who recalled a memory of when they ate too much subsequently spent almost twice as long helping the experimenter than those who recalled a neutral memory. They also showed greater motivation in helping by attempting and correctly solving more math questions than those in the neutral group. Because prosociality is one of the most common compensation methods for moral-wrong doing (e.g., Carlsmith and Gross, 1969; Stone et al., 1997; Ketelaar and Au, 2003; Ding et al., 2016), the presence of prosocial behavior after recalling an overeating memory indicates that people construe their overconsumption as a moral transgression. This interpretation is in line with the moral cleansing findings by Sheikh et al. (2013), who found an increased desire for cleansing products after recalling an overeating memory, which was driven by feelings of guilt and shame, signaling a sense of moral rule-breaking.

## Experiment 3

Unethical behaviors are not only compensated by prosocial acts. Religious practices such as self-flagellation after engaging in moral wrong-doing suggest that ethical violations also motivate self-punishment as a form of moral restitution (Coveney, 2006). Experimental research by Bastian et al. (2011) found that recalling a memory of socially excluding another person (vs. a neutral memory) led participants to spend more time on the painful cold pressor task, which consists of submerging one’s hand in ice-cold water (e.g., Snyder et al., 2005; Van Damme et al., 2008). Inbar et al. (2012) also showed that recalling a moral transgression (vs. a neutral memory) caused participants to administer stronger electric shocks to themselves. Furthermore, when participants were in the company of the victim of their transgression, they intended to administer stronger electric shocks to themselves than those left alone with the shock machine (Nelissen, 2011). Thus, self-punishment can be seen as aimed at restoring a threatened moral self-image.

**FIGURE 2:**
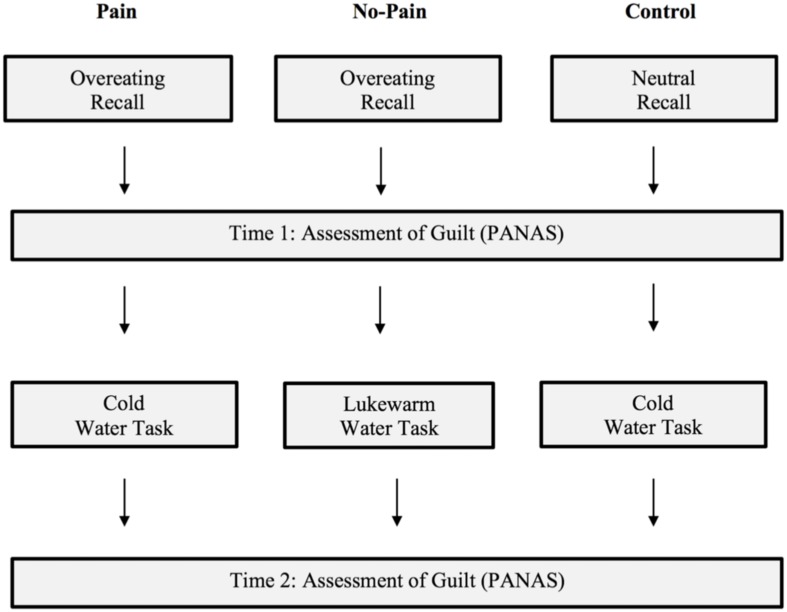
Graphic representation of the design of Experiment 3, as used by Bastian et al. (2011).

Experiment 3 tested whether recalling a time of excessive food consumption would similarly, lead to increased self-punishment. We closely followed the punishment task developed by Bastian et al. (2011), which involved three experimental conditions: two groups of participants recalled a time they overate (i.e., Pain and No-Pain Conditions), while another group of participants recalled their typical journey to work (i.e., Control Condition; see Figure 2 for experimental design). As the dependent variable participants engaged in the self-punishment task, for which they submerged their dominant hand under water to move paper clips between two containers, ostensibly as part of a task measuring physical precision. The Pain Condition completed the task in ice-cold water, whereas the No-Pain Condition did so in lukewarm water. The Control Condition completed the task in ice-cold water to allow comparison with the Pain Condition.

Guilt and shame resulting from the manipulation were measured by the Positive and Negative Affect Schedule (Watson et al., 1988). We also measured disgust, a moral emotion (Rozin et al., 1999; Schnall et al., 2008), and regret, an emotion often associated with non-moral failure (Ben-Ze’ev, 2000). Regret was included to assess whether overeating is seen by participants as a moral failure, or merely a violation of a social norm. The Positive and Negative Affect Schedule was administered immediately after the manipulation and then a second time after the self-punishment task to assess whether negative moral emotions were reduced by punishment.

Our main predictions were that, first, recalling an overeating memory would result in increased levels of guilt and shame. We therefore, compared guilt and shame reports of participants in the two overeating groups (Pain and No-Pain) with the Control Condition. Second, we examined subsequent self-punishment in the form of time spent in ice-cold water for the Pain and Control Conditions. Third, to test the prediction that self-punishment would decrease reported guilt and shame, we compared guilt and shame after the recall task (Time 1) to guilt and shame after the self-punishment task (Time 2) for the Pain Condition (involving ice-cold water) and the No-Pain Condition (involving lukewarm water). Furthermore, as in the earlier studies, we included the restrained eating subscale of the Dutch Eating Behavior Questionnaire (van Strien et al., 1986) to test whether guilt and shame, as well as self-punishment, responses to overeating recall are moderated by individual differences in restrained eating.

### Methods

#### Participants

A total of 67 female participants recruited through mailing lists, bulletin boards, and online participated in exchange for £3. One participant was removed due to not following instructions. The mean age was 23.03 years (*SD* = 5.38) and the sample was somewhat ethnically diverse: White (67.20%), East Asian (23%), Latin American (1.60%), Mixed ethnicity (4.90%), and no reported ethnicity (3.3%). An *a priori* decision was made regarding the sample size, with the number of participants per condition being roughly equal to Bastian et al.’s (2011) study (*n* = 19 to 23).

#### Procedure

A female experimenter tested participants individually in a laboratory for a study ostensibly on memory, emotions, sensory information and physical precision. They completed the same recall task as in Experiments 1 and 2, and the Positive and Negative Affect Schedule. Then participants received the water task and completed the Wong Baker Pain Scale, the Moral Self-Evaluation Scale, and the Dutch Eating Behavior Questionnaire subscale. Participants were then probed for suspicion by being asked about whether they had any ideas about the study purpose. None reported any insights regarding the hypotheses. Then they were debriefed, compensated, and thanked.

#### Manipulation

Participants were assigned to one of three conditions: Pain (overeating recall and ice-cold water immersion), No-Pain (overeating recall and lukewarm water immersion), or Control (neutral recall and ice-cold water immersion). Participants’ assignment to conditions was alternated throughout the study period.

#### Measures

##### Cold pressor task

Following Bastian et al. (2011), painful self-punishment was manipulated by having participants complete the cold pressor task (6–8°C) or a similar task with lukewarm water (19–21°C; see Figure 2 for design). The task consisted of a tub containing 100 marbles, and a tall glass bottle with a small opening into which participants could insert the marbles, all immersed in water in an insulated picnic cooler. Participants were instructed to insert individual marbles into the bottle without removing their hand from the water. For the cold pressor task participants were told that they should continue for as long as they wished, but that they could stop and withdraw their hand at any point. The water was kept cold with ice cubes, and the temperature was monitored using an underwater thermometer. To ensure the well-being of the participants they were stopped at 4 min. For the lukewarm water task participants were told that they should continue moving the marbles until the experimenter stopped them after 90 s. This cut-off point was employed by Bastian et al. (2011) as an approximation of how long the participants were expected to spend on average in the cold water condition. A fixed time was used because participants in the No-Pain Condition were not expected to discontinue the warm water task due to perceived discomfort, and it was nevertheless important to assess their level of guilt after the water task for comparison with the Pain Condition.

##### Positive and negative affect schedule (Watson et al., 1988)

To assess state guilt and shame the Positive and Negative Affect Schedule was administered, which is a commonly used measure of affect consisting of 20 items, with 10 items reflecting positive (e.g., “proud”; α = 0.86) and 10 items reflecting negative (e.g., “guilty”; α = 0.89) emotions. We added two more items to capture the negative emotions surrounding overeating: “disgusted” and “regretful.” Responses were indicated on a scale from 1 (“Slightly or not at all”) to 5 (“Extremely”).

##### Wong baker pain scale (Wong and Baker, 1988)

As in Bastian et al. (2011), pain level was assessed by asking participants, “How much hurt did you experience while holding your hand in the water?” rated from 1 (“No hurt”) to 5 (“Hurt worst”).

##### Moral self-evaluation scale

Based on Bastian et al. (2011), the degree to which participants perceived themselves and their actions to be immoral was measured by “I felt like what I did was wrong,” and “I was bad,” from 1(“Not at all”) to 7 (“Very much so”).

##### Dutch eating behavior questionnaire (van Strien et al., 1986)

The same eating restraint subscale as in Experiment 1 was used.

### Results

Following the analyses carried out by Bastian et al. (2011), the two groups recalling an overeating memory (Pain Condition and No-Pain Condition) were collapsed when comparing participants in the overeating (*n* = 43) versus control group (*n* = 24) on perceived immorality and experienced guilt and shame.

#### Manipulation Checks

To assess whether the overeating group showed increased perceptions of immorality compared to the control group, two independent samples *t*-test were carried out. As expected, participants who recalled an overeating memory (*M* = 3.33, *SD* = 1.60) perceived their behavior to be more wrong than those recalling a neutral memory (*M* = 1.22, *SD* = 0.43), *t* (50.52) = 8.06, *p* < 0.001, *d* = 1.61, 95% CI [1.59, 2.64]. The overeating group (*M* = 2.71, *SD* = 1.49) also perceived themselves to be more “bad” than the control group (*M* = 1.30, *SD* = 0.56), *t* (57.73) = 5.48, *p* < 0.001, *d* = 1.31, 95% CI [0.89, 1.29].

To check whether participants perceived the cold water in the Pain and Control conditions to be more painful than the No Pain Condition, an ANOVA was carried out. It confirmed an overall difference *F* (2, 62) = 35.86, *p* < 0.001, $\eta_{\text{p}}^{2}$ = 0.53, with Bonferroni corrected pairwise comparisons showing no difference between the Pain (*M* = 3.30, *SD* = 0.93) and Control conditions (*M* = 3.22, *SD* = 1.04), *p* = 0.77, but a significant difference both between Pain and No Pain (*M* = 1.26, *SD* = 0.45) conditions, *p* < 0.001, and the Control and No Pain conditions, *p* < 0.001.

#### Moral Emotions

To test whether the overeating memory induced guilt and shame, two independent samples *t*-tests were carried out. Participants recalling an overeating memory experienced more guilt (*M* = 2.02, *SD* = 1.28), *t* (63.87) = 3.02, *p* = 0.004, *d* = 0.66, 95% CI [0.25, 1.22], and shame (*M* = 1.86 *SD* = 1.29), *t* (46.99) = 3.57, *p* = < 0.001, *d* = 0.71, 95% CI [0.34, 1.21], than those recalling a neutral memory (guilt *M* = 1.29, *SD* = 0.68, shame *M* = 1.08, *SD* = 0.28). In addition, the moral emotion disgust, and the non-moral emotion regret were analyzed using independent *t*-tests. Disgust was significantly higher in the overeating group (*M* = 1.71, *SD* = 1.11) than the neutral group (*M* = 1.17, *SD* = 0.82), *t* (59.83) = 2.29, *p* = 0.03, *d* = 0.55, 95% CI [0.07, 1.03], but there was no difference in regret between the overeating (*M* = 1.93, *SD* = 1.26) and the neutral group (*M* = 1.54, *SD* = 0.88), *t* (61.11) = 1.46, *p* = 0.15, *d* = 0.34, 95% CI [−0.14, 0.92].

#### Self-Punishment

**FIGURE 3:**
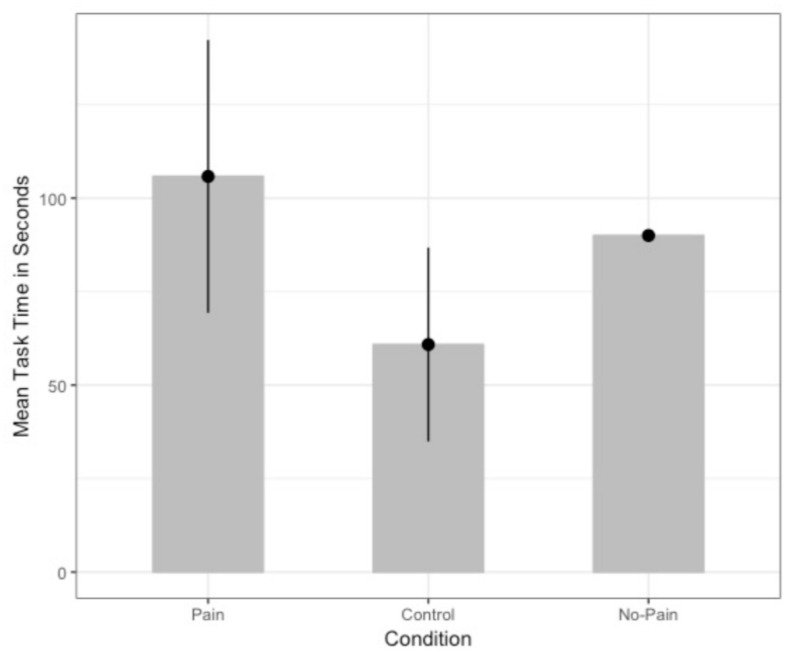
Mean time (in seconds) spent on the water task between the conditions in Experiment 3. The No-Pain Condition was set at 90 s. The means presented are the untransformed values to aid interpretation. Error bars represent 95% confidence intervals.

A general linear model (GLM) with condition as predictor of time for which the participant’s arm was immersed in water, controlling for water variations in temperature was conducted to test the primary hypothesis that overeating recall would lead to increased self-punishment. Only the two conditions who carried out the cold pressor task were compared. Residuals were significantly non-normal, *W* = 0.92, *p* = 0.002, but corrected with a log-transformation of the time variable, *W* = 0.98, *p* = 0.45. Figure 3 displays the untransformed means. Results with the log-transformed time variable revealed an overall effect of condition, *F* (1, 63) = 5.208, *p* = 0.03, $\eta_{\text{p}}^{2}$ = 0.12, 95% CI [−1.04, −0.11], with participants in the Pain Condition (*M* = 4.31, *SD* = 0.87) engaging in the self-punishment task for longer than those in the Control Condition (*M* = 3.79, *SD* = 0.75), *t* (46) = 2.85, *p* = 0.01, $\eta_{\text{p}}^{2}$ = 0.15. As a control test, an independent samples *t*-test showed that there was no significant difference in the time participants spent on the task in the Pain Condition and the No-Pain Condition (*M* = 4.45, *SD* = 0.00), *t* (41) = 0.87, *p* = 0.39, $\eta_{\text{p}}^{2}$ = 0.02, indicating that the time set for the No-Pain Condition was successful as a control time. Without including water temperature as covariate there also was a difference in task time between the two conditions, *F* (1, 46) = 4.97 *p* = 0.03, η^2^ = 0.10, 95% CI [1.66, 1.86].

#### Reduction of Moral Emotions

The following set of analyses were carried out to test whether the painful task (Pain condition) reduced guilt and shame more than the non-painful task (No Pain condition).

##### Guilt

A 2 (Pain vs. No-Pain) × 2 (Time 1 vs. Time 2) mixed ANOVA on guilt that participants reported less guilt from Time 1 (*M* = 2.05, *SD* = 1.28) to Time 2 (*M* = 1.17, *SD* = 1.44), *F* (1, 39) = 19.71, *p* < 0.001, $\eta_{\text{p}}^{2}$ = 0.34, 95% CI [1.38, 1.84]. However, this was not qualified by in interaction with Condition: participants in the Pain (Time 1 *M* = 2.05, *SD* = 1.30, Time 2 *M* = 1.18, *SD* = 1.16) and the No-Pain Condition (Time 1 *M* = 2.05, *SD* = 1.31, Time 2 *M* = 1.16, *SD* = 0.28) showing similar reductions in guilt, *F* (1, 39) = 0.01, *p* = 0.94, $\eta_{\text{p}}^{2}$ < 0.001.

##### Shame

A 2 (Pain vs. No-Pain) × 2 (Time 1 vs. Time 2) mixed ANOVA indicated a change in shame over time, such that participants reported a reduction in shame from Time 1 (*M* = 1.88, *SD* = 1.36) to Time 2 (*M* = 1.17, *SD* = 0.50), *F* (1, 39) = 10.20, *p* = 0.003, $\eta_{\text{p}}^{2}$ = 0.21, 95% CI [1.28, 1.76]. This was not qualified by an interaction with time: shame was not reduced more in the Pain (Time 1 *M* = 1.95, *SD* = 1.46, Time 2 *M* = 1.09, *SD* = 0.43) than in the No-Pain Condition (Time 1 *M* = 1.79, *SD* = 1.27, Time 2 *M* = 1.26, *SD* = 0.56), *F* (1, 39) = 0.60, *p* = 0.44, $\eta_{\text{p}}^{2}$ = 0.02.

#### Moderation: Restrained Eating Tendency

The prediction that high food restrainers spend a longer time on the cold pressor task, was assessed by moderation analyses. A 2 (Condition: Pain vs. Control) × continuous (restrained eating tendency) regression on the time spent on the cold pressor task showed that restrained eating tendency did not moderate time spent engaging in the painful task, *F* (1, 42) = 0.46, *p* = 0.50, $\eta_{\text{p}}^{2}$ = 0.11, indicating no individual differences on the main variable of interest.

### Discussion

The results confirmed that remembering excessive food consumption resulted in more guilt and shame and subsequently more self-inflicted pain than a neutral memory. These findings are consistent with previous literature showing that recalling a guilt-inducing event increased self-punishment (Bastian et al., 2011; Inbar et al., 2012). Guilt, shame and disgust, all moral emotions, were higher among those who tended to restrict their food intake. Regret, an emotion theorized to result from non-moral failures (Ben-Ze’ev, 2000) did not differ between conditions. The difference in feelings of guilt, shame and regret highlights that overeating is likely construed as a moral failure. Furthermore, participants were more likely to rate their behavior as wrong and themselves as bad after recalling an overeating event rather than a control event. This indicates that overeating is felt as a moral transgression and a break from what is deemed to be normative behavior for women (Basow and Kobrynowicz, 1993). These results were obtained across the sample, and were not moderated by tendencies to restrain food intake. Experiment 3 adds to the findings of Experiments 1 and 2, showing that overeating, as a moral failure, incurs the same emotional and behavioral consequences as traditional moral transgressions, most notably, prosocial behavior and self-punishment (Bastian et al., 2011; Nelissen, 2011; Inbar et al., 2012).

In contrast to Bastian et al. (2011) and Inbar et al. (2012), we did not find evidence for a guilt-reducing effect of self-inflicted pain. There are several possible explanations for this result. Higher levels of guilt (*M* = 2.37) were reported by participants in Bastian et al. (2011) manipulation condition—recalling an act of ostracism—than by those in the overeating group in this study (*M* = 2.03). Ostracizing may be more uniformly guilt-inducing and thus elicit less variance in affective responses, and this may account for the discrepancies in findings. Furthermore, Bastian et al’s (2011) Pain Condition had a higher baseline level of guilt (*M* = 2.53) than their No-Pain Condition (*M* = 2.21), allowing for a larger mean difference in the Pain Condition, whereas reported guilt in Experiment 3’s Pain vs. No-Pain Conditions were more similar. In comparison to the somewhat simpler task of moving paper clips adopted by Bastian and colleagues, it is also possible that engaging in the Experiment 3’s task required more skill and concentration (inserting small marbles into the narrow opening) and thus would itself have a guilt-reducing effect by, for example, providing a greater distraction from the overeating memory (Nolen-Hoeksema and Morrow, 1993). Finally, the sample size utilized by Bastian et al. (2011), on which we modeled the current study, was arguably small by current standards, therefore, the current work would benefit from additional replication.

## General Discussion

Three experiments demonstrated the emotional and behavioral consequences of the moralization of overeating. Based on the literature highlighting women’s moral experiences of self-sacrifice, Experiment 1 explored the effect of restrained eating practices and self-silencing, and found that relative to recalling a neutral life event, recalling a time when one “ate too much” increased participants’ self-silencing tendencies. Self-silencing therefore, could function to reaffirm one’s moral status in light of the guilt and shame of overeating.

Experiment 2 extended these findings using a measure of voluntary helping behavior. After recalling an overeating event, participants spent longer helping the experimenter by engaging in a mathematical questionnaire. As in Experiment 1, the findings indicate that overeating is perceived as a moral transgression, motivating participants to reestablish their moral status and compensate for their sense of wrongdoing. Finally, Experiment 3 highlighted the self-punishing consequences of failures in restrained eating practices. Participants who recalled an overeating memory reported increased guilt and shame as well as increased time inflicting pain on themselves, as a type of moral self-flagellation (Coveney, 2006). In contrast to Bastian et al. (2011), however, we did not find greater reduction of guilt and shame for participants doing the painful, self-punishing task of submerging their hand in ice-cold water compared to those who had the non-painful, lukewarm water. Overall, the studies reported in this paper extend previous findings on the moralization of eating practices to show the emotional and behavioral consequences of failures in normative food intake, particularly in terms of subsequent prosocial behaviors and self-punishment as efforts in moral compensation (e.g., Carlsmith and Gross, 1969; Ketelaar and Au, 2003; Bastian et al., 2011; Inbar et al., 2012; Ding et al., 2016).

The present research demonstrated moral compensation because of remembered excessive consumption, one side of moral self-regulation (Jordan et al., 2011). As previously mentioned, moral self-regulation also consists of increased licensing of unethical acts after good behavior (Blanken et al., 2015). If eating has moral connotations, and what is eaten has become part of the moral balancing act, behaving morally should license unhealthy food consumption, and eating healthy food should justify bad behavior. Indeed, Eskine (2013) reported that exposure to organic food (vs. comfort or neutral food) decreased participants’ intention to volunteer in a subsequent experiment (Eskine, 2013, although see also Moery and Calin-Jageman, 2016, for a smaller effect), in effect, giving them moral license to forgo this prosocial, volunteering behavior. Following the moral licensing effect (Merritt et al., 2010; Mullen and Monin, 2016), this finding suggests that organic food is perceived as “virtuous,” thereby reducing one’s need for moral self-reassurance. Thus, both “virtuous” and “sinful” food-related behaviors may affect one’s sense of morality and subsequent moral behaviors.

The studies recruited only women students as participants because of their greater tendency to moralize restrained eating (Sheikh et al., 2013) and to be concerned with weight and food intake (Striegel-Moore et al., 1989; Fayet et al., 2012), thereby limiting the generalizability of the findings to other populations, such as men, non-students and older age groups. There appears to be an increase in disordered eating patterns for both men and women (Hay et al., 2008; Mitchinson et al., 2014), making it important to investigate the ways in which men and other groups outside of our sample also moralize food intake.

The current research used recall of a past eating event in lieu of actual consumption, rendering us unable at this point to draw conclusions about whether similar consequences would occur after actual, *in vivo* failures in normative food consumption. Indeed, it is possible that participants in the overeating group remembered their most negative memory related to overeating, in line with research showing that recall is typically better for emotional events (e.g., Cahill and McGaugh, 1995), thus causing more distress than would occur during every-day failures in restrained eating. On the other hand, it has also been shown that emotions associated with negative events fade more quickly than those associated with positive events (see Walker et al., 2003 for a review), suggesting that the memories recalled by the overeating group would be less emotionally intense than actual failures in normative eating. Regardless, future research should test the impact of actual overeating and other failures in normative eating on subsequent moral compensatory behaviors, and it is possible that in such situations the effects are even more pronounced.

## Conclusion

Food consumption is an integral part of both everyday life and special occasions, and, as such, the ascribed meaning can have widespread consequences. Many religions and in secular practice alike consider food in moral terms, in which food consumption has a direct impact on one’s moral standing. From the results presented here, it is evident that the moral implications of food consumption can impact people’s behaviors, both toward others, in terms of increasing prosocial, helping actions, as well as toward themselves, by inflicting self-punishment. Furthermore, it is apparent that the moral significance of food in today’s society likely has a negative influence on women in particular, indicating that increased attention should be given to combat the ways in which the moral status of food consumption is advertised and portrayed in the media.

## Data Availability

The datasets generated for this study are available on request to the corresponding author.

## Ethics Statement

These studies were carried out in accordance with the recommendations of University of St Andrews “Teaching and Research Ethical Committee,” and University of Cambridge “Cambridge Psychology Research Ethics Committee,” with written informed consent from all subjects. All subjects gave written informed consent in accordance with the Declaration of Helsinki. The protocols were approved for Study 1 by the University of St Andrews “Teaching and Research Ethical Committee” and for Study 2 and 3 by University of Cambridge “Cambridge Psychology Research Ethics Committee.”

## Author Contributions

TS and SSh contributed to the design of Experiment 1. TS and SSc contributed to the design of Experiments 2 and 3. TS led on data collection, analysis, and drafting of the manuscript. SSh and SSc commented on and provided revisions, and edits on the manuscript.

## Conflict of Interest Statement

The authors declare that the research was conducted in the absence of any commercial or financial relationships that could be construed as a potential conflict of interest.

## Footnotes

1 For a detailed discussion of replications of this effect, and possible explanations for divergent findings, see Lee and Schwarz (2016).

2 We also conducted a linear regression, controlling for liking of mathematics, which showed the same result, β = 14, 24, *SE* = 3.39, *t* = 4.20, *p* < 0.001, *R*^2^ = 0.23, 95% CI [7.46, 21.04].
